# Antagonistic Activity of *Lactobacillus reuteri* Strains on the Adhesion Characteristics of Selected Pathogens

**DOI:** 10.3389/fmicb.2017.00486

**Published:** 2017-03-21

**Authors:** Tejinder P. Singh, Gurpreet Kaur, Suman Kapila, Ravinder K. Malik

**Affiliations:** ^1^Dairy Microbiology Division, National Dairy Research InstituteKarnal, India; ^2^Animal Biochemistry Division, National Dairy Research InstituteKarnal, India

**Keywords:** probiotics, *Lactobacillus reuteri*, adhesion, antagonistic activity, Caco-2 cells

## Abstract

Adhesion ability of probiotics is the key factor that decides their colonization in the gastrointestinal tract and potential to inhibit pathogens. Therefore, adhesion ability can be considered as a key determinant for probiotic efficacy. Presents study documents the antagonistic activity of viable/untreated, Lithium chloride (LiCl) treated or heat-killed forms of eight probiotic *Lactobacillus reuteri* strains on the adhesion characteristics of selected pathogens. All strains investigated were able to adhere to Caco-2 cells. *L. reuteri* strains tested were able to inhibit and displace (*P* < 0.05) the adhesion of *Escherichia coli* ATCC25922, *Salmonella typhi* NCDC113, *Listeria monocytogenes* ATCC53135, and *Enterococcus faecalis* NCDC115. The probiotic strain *L. reuteri* LR6 showed the strongest adhesion and pathogen inhibition ability among the eight *L. reuteri* strains tested. In addition, the abilities to inhibit and to displace adhered pathogens depended on both the probiotic and the pathogen strains tested suggesting the involvement of various mechanisms. The adhesion and antagonistic potential of the probiotic strains were significantly decreased upon exposure to 5 M LiCl, showing that surface molecules, proteinaceous in nature, are involved. The heat-killed forms of the probiotic *L. reuteri* strains also inhibited the attachment of selected pathogens to Caco-2 cells. In conclusion, *in vitro* assays showed that *L. reuteri* strains, as viable or heat-killed forms, are adherent to Caco-2 cells and are highly antagonistic to pathogens tested in which surface associated proteins play an important role.

## Introduction

Globally, the market of probiotics is growing faster as they have claimed to exert several health promoting effects, including interaction with the immune system, production of antimicrobial substances, enhancement of the mucosal barrier function and competition with enteropathogens for adhesion sites (Boesten and de Vos, 2008; Papadimitriou et al., 2015). There are numerous probiotic genera and species including lactobacilli and bifidobacteria which have been implicated in a number of health promoting functions that affect general health and well-being of the host. Adhesion is considered as a potential biomarker for selection of potential probiotics; as their colonization with extended transit time is extremely crucial for optimal expression of their general as well as specific physiological functions (Duary et al., 2011). Several reports have given special attention to the protective role of probiotics against enteropathogens and the underlying mechanisms (Salminen et al., 1998, 1999; Ouwehand et al., 2002; Ouwehand and Salminen, 2003; Collado et al., 2007). Some of these possible protective mechanisms include competition for nutrients and adhesion sites (Ouwehand and Salminen, 2003) or immune modulation (Schiffrin et al., 1997; Salminen et al., 1998). Thus, the probiotics intervention may provide significant protection against gastrointestinal infection and this would enhance human health.

Lactobacilli have been shown to possess surface adhesins similar to those on bacterial pathogens (Neeser et al., 2000). Several surface-located molecules such as lipoteichoic acid, lectin-like molecules and proteins have been identified as adhesins which interact with their specific receptors displayed on the host cell surface (Martinez et al., 2000; Beganović, 2008; Beganović et al., 2010). Due the importance of probiotics in the prevention of infections, the aim of this study was to assess the antagonistic properties of probiotic strains derived from breast fed human infant feces (Singh et al., 2012). Earlier, we reported that the cell surface proteins play an important role in probiotic activities of the *Lactobacillus reuteri* strains (Singh et al., 2016). In the present study, the probiotic *L. reuteri* strains were evaluated for their adhesion and abilities to exclude, displace and compete with selected pathogens using Caco-2 as an experimental model. These experiments were also conducted with LiCl treated and heat-killed forms of *L. reuteri* strains to check their functional interest and the importance of probiotic cell surface integrity.

## Materials and Methods

### Bacterial Strains and Culture Conditions

Eight *L. reuteri* strains viz., LR5, LR6, LR9, LR11, LR19, LR20, LR26, and LR34, of fecal origin were selected for this study. The *Lactobacillus* strains were grown in MRS broth (deMan, Ragosa and Sharp broth; Himedia, Mumbai, India) at 37°C for 18–24 h and maintained as glycerol stocks until further use. From the stock cultures, working cultures were prepared and were propagated twice prior to use by sub-culture in MRS broth. The bacterial pathogens used in this study were *Escherichia coli* ATCC25922, *Salmonella typhi* NCDC113, *Listeria monocytogenes* ATCC53135, *Enterococcus faecalis* NCDC115 which were maintained in BHI (Brain Heart Infusion) broth.

### Preparation of Probiotic *L. reuteri* Strains

#### Live Cells

The *Lactobacillus* strains were grown overnight (16–18 h) in MRS broth at 37°C and harvested at 5000 *g* for 10 min. The cells were washed twice in phosphate buffer saline (PBS) and OD_600_ was adjusted to 1.5 which corresponds to 10^9^ cfu/ml based on calibration curve performed.

#### LiCl Treatment

The *Lactobacillus* strains were harvested by centrifugation at 5000 *g* for 10 min, washed with sterile distilled water and then resuspended in 5 M LiCl for 30 min (Zhang et al., 2013). After LiCl treatment, the cells were washed twice in PBS and OD_600_ was adjusted to 1.5 which corresponds to 10^9^ cfu/ml.

#### Heat Killed Cells

The *Lactobacillus* strains were grown overnight (16–18 h) in MRS at 37°C and harvested by centrifugation at 5000 *g* for 10 min. Then, the cells were washed twice with PBS and OD_600_ was adjusted to 1.5 which corresponds to 10^9^ cfu/ml. The bacterial suspension was heat killed at 80°C for 10 min in a water bath and stored at -70°C until further use (Ouwehand et al., 2000).

### Caco-2 Cell Culture and Experiment Design

#### Caco-2 Cell Culture

The Caco-2 cell line was procured from the National Center of Cell Science, Pune, India. Cells were routinely grown in Dulbecco‘s modified eagle‘s minimal essential medium (DMEM; Sigma, USA), supplemented with 10% fetal bovine serum (FBS; Sigma, USA), 100 μg streptomycin per ml (Sigma, USA) and 100 U penicillin per ml (Sigma, USA) at 37°C in a 5% CO_2_ atmosphere. For adhesion and inhibition assays, Caco-2 monolayers were prepared in 6-well tissue culture plates. Cells were inoculated at a concentration of 7 × 10^4^ cells per well to obtain confluence and allowed to differentiate. The culture medium was changed on alternate days, and the last two media changes were without antibiotics.

#### *In vitro* Adherence Assay

A 1.0 ml aliquot of the bacterial suspension (viable, heat killed, and LiCl treated lactobacilli; 10^9^ cells) was added to confluent Caco-2 monolayer and incubated for 2 h in a 5% CO_2_ atmosphere. Following incubation, the Caco-2 monolayers were washed with sterile PBS (pH 7.4), Giemsa-stained and examined microscopically under oil immersion, as described previously by Duary et al. (2011).

#### Inhibition of Pathogen Adherence to Caco-2 Cells

The inhibition ability of viable/untreated, LiCl treated or heat-killed forms of *L. reuteri* strains against pathogens adherence was performed according to procedure described by Zhang et al. (2013) with some modifications. Three different protocols were followed to evaluate the ability of *L. reuteri* strains (viable, heat inactivated, and LiCl treated) to inhibit pathogen (*E. coli* ATCC25922, *L. monocytogenes* ATCC5313, *S. typhi* NCDC113, and *E. faecalis* NCDC115) adhesion to Caco-2 cells.

For competition assays, *Lactobacillus* (live, heat killed, and LiCl treated; approximately 10^8^–10^9^cfu/ml) and pathogens (approximately 10^7^cfu) were co-incubated with Caco-2 monolayer for 2 h. For exclusion assays, *Lactobacillus* (live, heat killed, and LiCl treated; approximately 10^8^–10^9^cfu/ml) was cultured with Caco-2 monolayer for 1 h. Following 1 h incubation, Caco-2 monolayer was washed three times with PBS (pH 7.4); pathogens (approximately 10^7^cfu) were added and incubated for another 1 h. For displacement assays, pathogens (approximately 10^7^cfu) were cultured with Caco-2 monolayer for 1 h, and then the *Lactobacillus* (live, heat killed, and LiCl treated) were added and cultured for another 1 h. The monospecies cultures of pathogenic bacteria were used as the controls.

In all the above treatments, non-adhered bacterial cells were removed by washing with PBS (pH 7.4). After washing, the Caco-2 cells were detached by addition of 0.25% (v/v) Trypsin-EDTA solution at 37°C for 5 min and the number of viable adhering *E. coli, L. monocytogenes, S. typhi*, and *E. feacalis* were determined by plating on EMB, PALCAM, XLD, and CA agar plates after serial dilutions, respectively.

### Statistical Analysis

The results for adhesion and pathogen inhibition are expressed as the mean ± SD of three independent experiments. Statistical analysis was done by StatGraphicPlus software. Data were subjected to a one-way analysis of variance (ANOVA) followed by a Tukey’s *post hoc* test. Differences were considered statistically significant when *P* < 0.05.

## Results

### Adhesion Assay

**FIGURE 1:**
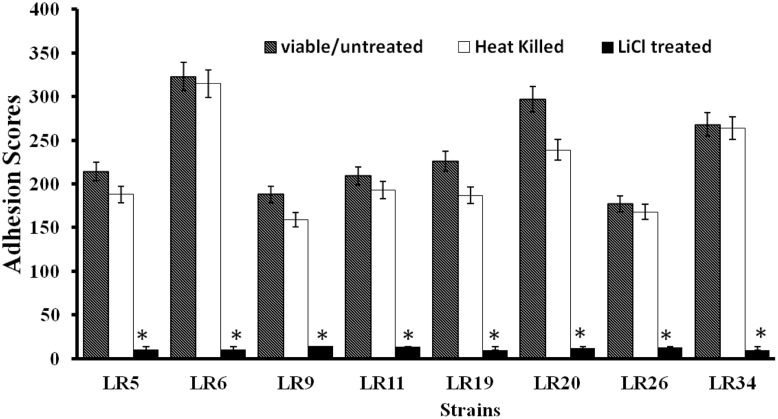
**Adhesion of differently treated *Lactobacillus reuteri* strains to Caco-2 cells.** ^∗^Significantly different (*P* < 0.05) from the untreated control.

All the *L. reuteri* strains adhered to Caco-2 cells albeit at different levels. However, on comparative evaluation, *L. reuteri* strains LR6, LR20, and LR34 were found to be the most adhesive strains based on their respective adhesion scores, with LR6 being the most adhesive strain among all the strains tested. The adhesion score for other strains tested, i.e., LR5, LR9, LR11, LR19, and LR26 differed significantly. All the *L. reuteri* test strains were found to be highly adhesive (>100 bacteria/20 microscopic fields) when assessed in Caco-2 cell lines. In comparison, it was also observed that the heat inactivation and LiCl treatment had a marked effect on the adhesion ability of the strains as the adhesion of the strains was significantly (*P* < 0.05) reduced as shown in **Figure 1**.

### Competition Assay

Competition assay explained the ability of probiotic strains to compete with pathogens for the adhesion site on epithelial cells. Among the *L. reuteri* strains tested; LR6, LR9, and LR11 exhibited the maximum inhibition of *E. coli* ATCC25922. For *S. typhi* NCDC113, the strains mainly *L. reuteri* LR6, LR9, LR11, LR19, and LR26 showed the maximum inhibition. Among the tested probiotic *L. reuteri* strains; LR5, LR6, LR9, LR20, and LR34 inhibited the adhesion of *L. monocytogenes* ATCC53135 to Caco-2 cells to significant (*P* < 0.05) levels. For *E. feacalis* NCDC115, the significant inhibition of adhesion to Caco-2 cells was seen for the strain LR6 and LR9. From the results of competitive assay shown in **Table 1**, we can conclude that the strain LR6 is the most competitive probiotic strain which can compete strongly with the selected pathogens for the adhesion to epithelial cells.

**Table 1:** Competence between pathogens and differently treated probiotic *Lactobacillus reuteri* strains to adhere to Caco-2 cells.

Strain	*E. coli* ATCC25922			*S. typhi* NCDC113			*L. monocytogenes* ATCC53135			*E. faecalis* NCDC115		
				
	Viable untreated	Heat killed	LiCl treated	Viable untreated	Heat killed	LiCl treated	Viable untreated	Heat killed	LiCl treated	Viable untreated	Heat killed	LiCl treated
LR5	85.0 ± 1.0^bcy^	86.5 ± 2.0^cdy^	91.2 ± 1.2^ax^	80.0 ± 1.5^bxy^	76.5 ± 2.7^by^	81.2 ± 1.2^axy^	67.0 ± 1.0^fz^	84.3 ± 1.2^cdy^	88.8 ± 0.6^bcx^	82.5 ± 1.3^aby^	85.6 ± 0.9^cy^	89.8 ± 1.2^ax^
LR6	59.5 ± 2.3^fy^	62.4 ± 1.3^gy^	89.4 ± 1.2^ax^	47.5 ± 1.7^fy^	42.6 ± 0.8^fz^	88.4 ± 1.6^bx^	56.0 ± 0.9^gz^	79.6 ± 0.9^fgy^	87.5 ± 1.0^cdx^	60.5 ± 1.0^ez^	74.2 ± 1.7^dy^	89.3 ± 1.7^ax^
LR9	67.5 ± 1.2^ez^	74.3 ± 1.4^fy^	83.7 ± 1.8^bx^	55.0 ± 0.4^ey^	57.5 ± 1.0^ey^	79.6 ± 2.8^ax^	69.5 ± 1.0^ez^	77.8 ± 0.9^gy^	88.2 ± 1.0^cdx^	69 ± 1.4^dz^	77.2 ± 1.0^dy^	87.3 ± 2.0^ax^
LR11	72.0 ± 1.5^dy^	82.3 ± 1.9^ex^	83.9 ± 1.3^bax^	61.0 ± 0.6^dy^	62.8 ± 1.0^dy^	81.5 ± 1.3^ax^	89.5 ± 1.0^ax^	91.7 ± 1.1^ax^	91.9 ± 0.8^ax^	77 ± 1.7^cy^	84.7 ± 1.4^cx^	83.9 ± 1.6^bx^
LR19	82.5 ± 2.1^cy^	84.2 ± 0.9^dey^	91.3 ± 1.8^ax^	75.0 ± 0.1^cy^	72.4 ± 1.3^cy^	88.7 ± 3.0^bx^	78.0 ± 0.9^cz^	83.1 ± 0.9^dey^	90.7 ± 0.9^abx^	77.5 ± 1.1^cy^	86.8 ± 1.5^cx^	89.1 ± 0.6^ax^
LR20	87.5 ± 1.7^aby^	91.4 ± 1.8^abxy^	93.2 ± 1.5^ax^	85.0 ± 2.0^ay^	81.1 ± 1.3^ay^	92.3 ± 2.8^bx^	71.5 ± 1.2^dey^	87.7 ± 1.1^bx^	87.4 ± 0.8^cdx^	85.5 ± 1.2^ay^	90.7 ± 1.7^abx^	88.4 ± 1.0^axy^
LR26	89.5 ± 1.0^ax^	93.3 ± 1.1^ax^	90.8 ± 2.6^ax^	75.0 ± 2.4^cy^	78.2 ± 1.8^aby^	89.1 ± 1.9^bx^	86.0 ± 0.8^by^	91.4 ± 1.1^ax^	88.6 ± 0.9^cdxy^	81.5 ± 1.7^bz^	91.1 ± 1.5^ax^	86.8 ± 1.1^aby^
LR34	86.0 ± 1.2^by^	89.5 ± 1.7^bcxy^	92.1 ± 0.9^ax^	80.0 ± 2.3^by^	75.7 ± 2.1^bc^	90.3 ± 1.3^bx^	72.5 ± 1.1^dy^	86.4 ± 0.7^bcx^	86.7 ± 0.9^dx^	79 ± 2.5^bcy^	87.6 ± 1.2^bcx^	87.5 ± 1.3^ax^


*Data are adherence ratio of pathogen to Caco-2 cells = (test/control) × 100%, shown as mean ± standard deviation of three independent experiments. ^abcdefg^ Different symbol means statistically significant difference (*P* < 0.05) within the same column. ^xyz^ Different symbol means statistically significant difference (*P* < 0.05) within the same row between the treatments.*

In competition inhibition assay, it was observed that heat inactivation decreases the ability of *L. reuteri* strains to compete with selected pathogens for adhesion to Caco-2 cells as compared with their untreated/live forms. The inhibition ability of the heat inactivated forms of *L. reuteri* strains showed the variability in results ranging from 6.7 ± 1.13% to 37.6 ± 1.07% for *E. coli* ATCC25922, 18.9 ± 1.32% to 57.4 ± 2.32% for *S. typhi* NCDC113, 8.3 ± 0.61% to 20.4 ± 1.17% for *L. monocytogenes* ATCC53135, and 8.9 ± 1.27% to 25.8 ± 1.46% for *E. feacalis* NCDC115 as shown in **Table 1**. For heat inactivated forms, the strains LR6, LR9, and LR11 showed the maximum inhibition to adhesion of *S. typhi* NCDC113. In case of *E. coli* ATCC25922, the maximum inhibition was exhibited by heat inactivated forms of LR6, LR9, LR11, and LR19, respectively. The strain LR6 also showed the highest inhibition of *L. monocytogenes* ATCC53135 and *E. feacalis* NCDC115 adhesion to Caco-2 cells. From the results given in **Table 1**, it is also evident that the heat inactivated forms of *L. reuteri* strains were able to compete with pathogens as the results on comparison were found statistically insignificant to their viable forms. However, the ability of the *L. reuteri* strains to compete with pathogens assayed for adhesion site on Caco-2 decreases significantly (*P* < 0.05) on LiCl treatment (meant for removal of surface proteins) as depicted in **Table 1**.

### Displacement Assay

Displacement assay exhibits the potential of the probiotic strains to remove/displace the already adhered pathogen from the epithelial cells. The data depicted that amongst the *L. reuteri* strains tested, the strain LR6 showed maximum inhibition of *E. coli* ATCC25922, *S. typhi* NCDC113, *L. monocytogenes* ATCC53135, and *E. feacalis* NCDC115. After 5 M LiCl treatment, the displacement ability of *L. reuteri* strains against test pathogens were significantly reduced (*P* < 0.05), shown in **Table 2**.

**Table 2:** Displacement of pathogens adhering to Caco-2 cells by differently treated probiotic *L. reuteri* strains.

Strain	*E. coli* ATCC25922			*S. typhi* NCDC113			*L. monocytogenes* ATCC53135			*E. faecalis* NCDC115		
				
	Viable untreated	Heat killed	LiCl treated	Viable untreated	Heat killed	LiCl treated	Viable untreated	Heat killed	LiCl treated	Viable untreated	Heat killed	LiCl treated
LR5	82.5 ± 1.3^cy^	88.5 ± 2.4^cdx^	88.2 ± 0.6^bx^	75.0 ± 1.7^cy^	88.4 ± 2.2^abx^	80.2 ± 1.7^cdy^	85.5 ± 1.1^bx^	88.8 ± 0.9^bcx^	88.5 ± 1.5^bcx^	69.5 ± 0.9^dy^	85.8 ± 1.4^cx^	85.4 ± 1.3^dx^
LR6	59.5 ± 1.7^ez^	79.6 ± 1.1^fy^	93.2 ± 1.2^ax^	49.5 ± 2.5^fz^	66.4 ± 0.9^dy^	87.4 ± 1.5^abx^	59.5 ± 0.9^ez^	78.3 ± 0.9^ey^	89.3 ± 1.0^bcx^	64.5 ± 1.3^ez^	76.4 ± 1.3^ey^	89.3 ± 0.6^bx^
LR9	71.5 ± 1.5^dz^	83.3 ± 0.6^ey^	87.3 ± 1.5^bx^	61.5 ± 1.0^ey^	79.4 ± 2.1^cx^	77.3 ± 2.0^dx^	81.1 ± 0.9^cy^	87.5 ± 1.0^cx^	87.9 ± 0.8^cx^	71 ± 0.7^dz^	84.2 ± 1.1^cy^	88.3 ± 1.4^bcx^
LR11	87.5 ± 1.7^bxy^	91.5 ± 0.8^bx^	86.3 ± 2.7^by^	85.0 ± 1.7^by^	91.5 ± 1.6^ax^	83.6 ± 2.0^bcy^	86.5 ± 0.7^aby^	90.7 ± 0.9^abx^	89.4 ± 1.0^bcx^	81 ± 0.6^bz^	90.4 ± 1.2^abx^	86.4 ± 1.2^cdy^
LR19	74.5 ± 2.1^dz^	89.9 ± 0.9^bcx^	78.5 ± 0.7^cy^	70.0 ± 2.0^dy^	90.7 ± 1.3^ax^	87.8 ± 0.9^ax^	69.0 ± 0.9^dz^	87.5 ± 1.0^cx^	84.9 ± 0.6^dy^	86 ± 1.2^ax^	88.7 ± 0.9^bx^	78.9 ± 1.2^ey^
LR20	93.5 ± 1.2^ax^	95.1 ± 0.8^ax^	94.1 ± 0.8^ax^	90.0 ± 1.2^ax^	90.8 ± 0.6^ax^	90.3 ± 2.1^ax^	88.0 ± 1.0^ay^	91.8 ± 1.0^ax^	92.4 ± 0.7^ax^	82 ± 0.7^bz^	89.6 ± 1.0^aby^	93.6 ± 1.6^ax^
LR26	83.5 ± 2.1^cy^	86.7 ± 1.8^dy^	91.8 ± 0.7^ax^	75.1 ± 2.0^cy^	86.4 ± 2.0^bx^	89.1 ± 1.2^ax^	81.0 ± 1.0^cy^	89.6 ± 0.9^bcx^	91.8 ± 0.9^ax^	88 ± 1.0^ax^	91.7 ± 1.9^ax^	90.7 ± 1.3^bx^
LR34	73 ± 1.2^dz^	83.5 ± 0.8^ey^	92.3 ± 0.5^ax^	67.5 ± 2.8^dz^	80.7 ± 2.4^cy^	89.4 ± 1.9^ax^	88.5 ± 0.9^ax^	90.7 ± 1.0^abx^	90.3 ± 0.7^abx^	74.5 ± 1.1^cy^	89.5 ± 1.0^abx^	89.3 ± 1.0^bx^


*Data are adherence ratio of pathogen to Caco-2 cells = (test/control) × 100%, shown as mean ± standard deviation of three independent experiments. ^abcdef^ Different symbol means statistically significant difference (*P* < 0.05) within the same column. ^xyz^ Different symbol means statistically significant difference (*P* < 0.05) within the same row between the treatments.*

The heat inactivated forms of *L. reuteri* strains showed reduced ability to displace the tested pathogens as compared to their untreated/viable forms. The heat inactivated forms of *L. reuteri* strains showed the variability in results for percentage displacement ranging from 6.5 ± 1.16% to 40.5 ± 0.99% for *E. coli* ATCC25922, 10 ± 0.16% to 50.5 ± 2.52% *S. typhi* NCDC113, 11.5 ± 1.03% to 40.5 ± 1.03% *L. monocytogenes* ATCC53135, and 12 ± 0.93% to 35.5 ± 0.89% for *E. feacalis* NCDC115 as shown in **Table 2**. For heat inactivated forms, the strains LR6 and LR9 showed the maximum displacement for *S. typhi* NCDC113. In case of *E. coli* ATCC25922, the maximum displacement was exhibited by heat inactivated forms of LR6. The strain LR6 also showed the highest inhibition of *L. monocytogenes* ATCC53135 and *E. feacalis* NCDC115 to Caco-2 cells.

### Exclusion Assay

Exclusion assay explains that once the adhesion site is occupied by the probiotic bacteria it becomes unavailable for pathogen. It is evident from the results that the tested strains LR5, LR6, LR9, LR20, and LR26 were able to exclude *E. coli* ATCC25922 adhesion to significant levels. The significant reduction in *E. coli* ATCC25922 adhesion to Caco-2 cells was observed for LR5, LR6, LR9, LR20, and LR26. In case of *S. typhi* NCDC113, the maximum exclusion was showed by strains LR5, LR6, LR9, LR19, and LR26. On the other hand, only LR6 showed the maximum exclusion of *L. monocytogenes* ATCC53135 from adhesion to caco-2 cells. Similarly, LR6 and LR11 were the only strains which were able to exclude the *E. feacalis* NCDC115 to significant levels. The data is depicted in **Table 3**.

**Table 3:** Exclusion of pathogens from adhesion to Caco-2 cells by differently treated probiotic *L. reuteri* strains.

Strain	*E. coli* ATCC25922			*S. typhi* NCDC113			*L. monocytogenes* ATCC53135			*E. faecalis* NCDC115		
				
	Viable untreated	Heat killed	LiCl treated	Viable untreated	Heat killed	LiCl treated	Viable untreated	Heat killed	LiCl treated	Viable untreated	Heat killed	LiCl treated
LR5	67.5 ± 1.0^dy^	87.3 ± 1.2^ax^	89.5 ± 1.0^cdx^	72.5 ± 2.1^az^	81.7 ± 2.5^axy^	83.5 ± 3.6^bcx^	88.5 ± 1.1^bx^	89.2 ± 0.9^bcx^	89.5 ± 1.5^dex^	71.5 ± 1.3^dey^	83.9 ± 1.2^fx^	85.9 ± 1.1^ax^
LR6	56 ± 1.1^fz^	82.2 ± 1.0^by^	93.1 ± 1.2^abx^	49 ± 2.0^dz^	62.2 ± 2.6^by^	91.6 ± 1.9^ax^	61.5 ± 0.9^ez^	86.3 ± 0.9^dey^	92.1 ± 1.0^bcx^	68.5 ± 1.3^ez^	84.6 ± 1.2^efy^	91.2 ± 2.6^ax^
LR9	67.5 ± 1.5^dy^	87.4 ± 0.4^ax^	87.4 ± 0.8^dx^	55 ± 1.2^cz^	86.2 ± 1.8^ax^	78.4 ± 2.4^dy^	66.5 ± 0.9^dy^	88.4 ± 1.0^cdx^	85.8 ± 0.8^fx^	86.5 ± 1.0^bx^	89.5 ± 0.9^bx^	88.5 ± 1.3^ax^
LR11	80.5 ± 0.8^ay^	88.4 ± 0.8^ax^	88.4 ± 1.1^cdx^	75 ± 1.5^ay^	84.8 ± 3.3^ax^	83.4 ± 1.7^bcx^	94 ± 0.7^ax^	95.3 ± 0.9^ax^	95.4 ± 1.0^ax^	63.5 ± 1.0^fy^	89.3 ± 0.5^bcx^	89.4 ± 0.8^ax^
LR19	74 ± 0.4^bz^	86.6 ± 0.8^ay^	93.1 ± 1.3^abx^	62.5 ± 2.5^by^	82.6 ± 2.4^ax^	81.3 ± 1.6^cdx^	88 ± 0.9^by^	89.7 ± 1.0^bcxy^	91.3 ± 0.6^bcdx^	85 ± 1.0^by^	86.6 ± 1.0^dey^	91.3 ± 0.9^ax^
LR20	69.5 ± 0.9^cdz^	86.5 ± 0.4^ay^	90.7 ± 0.9^bcx^	77 ± 2.2^ay^	85.6 ± 2.3^ax^	90.7 ± 2.1^ax^	73 ± 1.0^cy^	85.7 ± 1.0^ex^	87.9 ± 0.7^efx^	79.5 ± 1.3^cz^	86.5 ± 0.9^dey^	89.7 ± 0.8^ax^
LR26	59.5 ± 1.1^ez^	83.1 ± 1.2^by^	93.7 ± 1.1^ax^	61 ± 3.1^by^	81.3 ± 1.6^ax^	83.7 ± 2.0^bcx^	88 ± 1.0^by^	90.7 ± 0.9^by^	93.7 ± 0.9^abx^	91 ± 2.5^ax^	92.8 ± 1.2^ax^	92.7 ± 1.2^ax^
LR34	81 ± 0.8^az^	87.3 ± 0.8^ay^	92.2 ± 1.2^abx^	76.5 ± 3.0^ay^	81.7 ± 2.0^axy^	88.2 ± 2.1^abx^	72.5 ± 0.9^cz^	87.7 ± 1.0^cdey^	91.3 ± 0.7^bcdx^	74.5 ± 0.9^bz^	86.9 ± 1.1^cdey^	90.6 ± 1.1^ax^


*Data are adherence ratio of pathogen to Caco-2 cells = (test/control) × 100%, shown as mean ± standard deviation of three independent experiments. ^abcdef^ Different symbol means statistically significant difference (*P* < 0.05) within the same column. ^xyz^ Different symbol means statistically significant difference (*P* < 0.05) within the same row between the treatments.*

The heat inactivated forms of probiotic strains showed significantly reduced exclusion of the pathogens from Caco-2 cells when compared with their untreated viable forms. The exclusion activity of the heat inactivated forms of *L. reuteri* strains also showed the variability in results ranging from 11.6 ± 1.06% to 17.8 ± 1.36% for *E. coli* ATCC25922, 13.8 ± 2.25% to 37.8 ± 2.70% *S. typhi* NCDC113, 4.7 ± 0.75% to 14.3 ± 1.07% *L. monocytogenes* ATCC53135, 7.2 ± 1.06% to 16.1 ± 1.27% for *E. feacalis* NCDC115. The strains LR6, LR9, LR20, and LR34 showed the maximum exclusion of *L. monocytogenes* ATCC53135 *in vitro*. For *E. coli* ATCC25922 and *S. typhi* NCDC113, the maximum exclusion was reported for strain LR6. The strains LR5, LR6, LR19, LR20, and LR34 showed the highest exclusion for *E. feacalis* NCDC115 *in vitro*. However, the ability of the *L. reuteri* strains to exclude pathogens tested decreased significantly (*P* < 0.05) on LiCl treatment (meant for removal of surface proteins) (**Table 3**).

## Discussion

Probiotics efficacy is highly dependent on their survival and persistence in gastrointestinal tracts. Therefore, adhesion ability can be considered as a standard biomarker for selecting a potential probiotic (Duary et al., 2011). In the present investigation, we evaluated eight probiotic strains of *L. reuteri*, previously isolated from breast fed infant feces (Singh et al., 2012), for their potential to adhere Caco-2 cells. The results pertaining to adhesion were recorded in terms of number of bacteria adhering to Caco-2 cell line. On comparative evaluation based on adhesion score, *L. reuteri* strains LR6, LR20, and LR34 were found to be the most adhesive strains. Adhesion score for all the *L. reuteri* strains were more than 100 and, therefore, can be regarded as a strongly adhesive to Caco-2 cell lines as per the classification by Jacobsen et al. (1999). Also, the variation observed in adhesion abilities of *L. reuteri* strains suggests that the trait varies among probiotic strains. This is in complete agreement with the other researchers who also reported that the probiotics ability to adhere is very much strain, species and genus specific (Collado et al., 2007).

Several studies have reported that probiotics compete with pathogens for the adhesion sites, as both probiotics and pathogens possess similar kind of adhesins on their surfaces. Also, the inhibition specifically depends on the probiotic strains and pathogens used as well as the methods of assessment (Chen et al., 2007; Gueimonde et al., 2007). In this study, the probiotic *L. reuteri* strains were evaluated for their abilities to exclude, compete and displace selected pathogens using Caco-2 as an experimental model. The pathogen adhesion inhibition by probiotic *L. reuteri* strains showed a high variability and indicated that it was clearly a strain dependent property. The *L. reuteri* strains tested did not show the same level of inhibition capacity against the pathogens, but they efficiently inhibited the adhesion of pathogenic bacteria to Caco-2 cell in all three assays. The strain LR6 with highest adhesion ability generally showed much higher inhibition of pathogen adhesion to Caco-2 cells, indicating that the pathogen inhibition capacity of probiotic strains may be related to their adhesion ability. Similarly, other workers have also reported the competitive exclusion of enteropathogens by bifidobacteria and lactobacilli (Bernet et al., 1993; Forestier et al., 2001; Lee et al., 2003; Collado et al., 2005, 2007; Weizman et al., 2005; Pham et al., 2009; Wine et al., 2009; Zhang et al., 2013). Meanwhile, the pathogen inhibition ability of the *L. reuteri* strains did not correlate with the adhesive ability of the strains. In our results, the profile of competition, exclusion and displacement of pathogens by *L. reuteri* strains were different confirming that the mechanisms of competition, exclusion and displacement might be different. Therefore, it was believed that the differences in competitive exclusion between the strains correlate with the variations in their adhesion ability, possibly due to differences in their surface characteristics. This suggests that the mechanism involved in inhibition is complicated and many factors may be involved.

Generally, adhesion involves the interaction between bacterial associated molecular patterns such as; lipoteichoic acid (Granato et al., 1999), surface layer protein (Chen et al., 2007; Johnson-Henry et al., 2007), peptidoglycan (Van Tassell and Miller, 2011) and their pattern recognition receptors on the host epithelial cells. The surface associated proteinaceous components mediating bacterial adhesion to intestinal epithelial cells have been demonstrated for many *Lactobacillus* species (Rojas et al., 2002; Roos and Jonsson, 2002; Frece et al., 2005; Chen et al., 2007). In the present study, a significant (*P* < 0.05) difference was observed on comparing the adhesion ability of untreated and LiCl treated forms of *L. reuteri* strains, suggesting the importance of the surface associated proteins in adhesion. Also, the ability of the *L. reuteri* strains to displace, compete and exclude the pathogens from adhesion to caco-2 cells was significantly (*P* < 0.05) decreased on LiCl treatment. The results are in complete agreement with other workers who reported reduction in binding and adhesion ability of lactobacilli on removal/disruption of surface associated proteins (Sillanpää et al., 2000; Buck et al., 2005; Frece et al., 2005; Chen et al., 2007; Johnson-Henry et al., 2007; Wang et al., 2008; Li et al., 2011; Zhang et al., 2013).

By definition probiotics should be viable in order to exert health benefits. Many researchers have suggested that certain probiotic effects can also be obtained with non-viable probiotics (Ouwehand and Salminen, 1998). Evidences also suggested that non-viable probiotics are less effective which may be attributed to their reduced binding ability than viable probiotics (Conge et al., 1980; De Simone et al., 1987; Kato et al., 1994; Kaila et al., 1995; Perdigon et al., 1995). In this study, a significant reduction in the adhesion and pathogen inhibition abilities of the probiotic *L. reuteri* strains was observed in heat inactivated forms compared to their viable forms. This suggests that heat treatment inactivate the micro-organisms and also alters their physicochemical properties (El-Nezami et al., 1998). The reduction of adhesion and pathogen inhibition can be explained by the heat sensitive proteinaceous nature of the molecules involved. In contrast, Tareb et al. (2013) reported that heat-killed forms of both *Lb. rhamnosus* 3698 and *Lb. farciminis* 3699 exhibited higher adhesion and higher pathogen exclusion potential.

Probiotics intervention is more cost effective and natural approach to preserve intestinal homeostasis and restore the pathogenesis related dysbiosis than antibiotics. The results of this study demonstrate that probiotic strains of *L. reuteri* tested can exclude, displace and compete with enteropathogens. However, it is important to take into account that these processes studied are highly specific to probiotic and pathogenic strains. This study indicates that strong adhesion ability means greater inhibition activity for probiotic lactobacilli against pathogen, in which surface associated proteins play an important role which further need to be identified and studied. This study also supports the need for further investigations to demonstrate the potential benefits of *L. reuteri* strains, particularly strain LR6, live or heat-killed, in the food chain.

## Author Contribution

All the authors listed, have made substantial, direct and intellectual contribution to the work, and approved it for publication.

## Conflict of Interest Statement

The authors declare that the research was conducted in the absence of any commercial or financial relationships that could be construed as a potential conflict of interest.

## References

[B1] Beganović J. (2008). *Application of Proteomics and Other Molecular Methods in the Characterization of Functionality of the Probiotic Bacteria.* Ph.D. dissertation, University of Zagreb, Zagreb.

[B2] Beganović J. Guillot A. van de Guchte M. Jouan A. Gitton C. Loux V. (2010). Characterization of the insoluble proteome of *Lactococcus lactis* by SDS-PAGE LC-MS/MS leads to the identification of new markers of adaption of the bacteria to the mouse digestive tract. *J. Proteome Res.* 9 677–688. 10.1021/pr9000866 20000844

[B3] Bernet M. F. Brassart D. Nesser J. R. Servin A. L. (1993). Adhesion of human bifidobacterial strains to cultured human intestinal epithelial cells and inhibition of enterophatogen- cell interactions. *Appl. Environ. Microbiol.* 59 4121–4128. 8285709 10.1128/aem.59.12.4121-4128.1993 PMC195875

[B4] Boesten R. J. de Vos W. M. (2008). Interactomics in the human intestine: lactobacilli and bifidobacteria make a difference. *J. Clin. Gastroenterol.* 42 S163–S167. 10.1097/MCG.0b013e31817dbd62 18685514

[B5] Buck B. L. Altermann E. Svingerud T. Klaenhammer T. R. (2005). Functional analysis of putative adhesion factors in *Lactobacillus acidophilus* NCFM. *Appl. Environ. Microbiol.* 71 8344–8351. 10.1128/AEM.71.12.8344-8351.2005 16332821 PMC1317474

[B6] Chen X. Y. Xu J. J. Shuai J. B. Chen J. S. Zhang Z. F. Fang W. H. (2007). The S-layer proteins of *Lactobacillus crispatus* strain ZJ001 is responsible for competitive exclusion against *Escherichia coli* O157:H7 and *Salmonella typhimurium*. *Int. J. Food Microbiol.* 115 307–312. 10.1016/j.ijfoodmicro.2006.11.007 17289201

[B7] Collado M. C. Gueimonde M. Hernández M. Sanz Y. Salminen S. (2005). Adhesion of selected *Bifidobacterium* strains to human intestinal mucus and the role of adhesion in enteropathogen exclusion. *J. Food Prot.* 68 2672–2678. 10.4315/0362-028X-68.12.2672 16355841

[B8] Collado M. C. Surono I. Meriluoto J. Salminen S. (2007). Indigenous dadih lactic acid bacteria: cell-surface properties and interactions with pathogens. *J. Food Sci.* 72 M89–M93. 10.1111/j.1750-3841.2007.00294.x 17995806

[B9] Conge G. A. Gouache P. Desormeau-Bedot J. P. Loisillier F. Lemonnier D. (1980). Comparative effects of a diet enriched in live or heated yogurt on the immune system of the mouse. *Reprod. Nutr. Dev.* 20 929–938. 10.1051/rnd:19800603 7349461

[B10] De Simone C. Vesely R. Negri R. Bianchi S. B. Zanzoglu S. Cilli A. (1987). Enhancement of immune response of murine Peyer’s patches by a diet supplemented with yoghurt. *Immunopharmacol. Immunotoxicol.* 9 87–100. 10.3109/08923978709035203 3502471

[B11] Duary R. K. Rajput Y. S. Batish V. K. Grover S. (2011). Assessing the adhesion of putative indigenous probiotic lactobacilli to human colonic epithelial cells. *Ind. J. Med. Res.* 134 664–671. 10.4103/0971-5916.90992 PMC3249965 22199106

[B12] El-Nezami H. KankaanpaÈaÈ P. Salminen S. Ahokas J. (1998). Physico-chemical alterations enhance the ability of dairy strains of lactic acid bacteria to remove aflatoxin from contaminated media. *J. Food Prot.* 61 466–468. 10.4315/0362-028X-61.4.466 9709211

[B13] Forestier C. De Champs C. Vatoux C. Joly B. (2001). Probiotic activities of *Lactobacillus casei* rhamnosus: in vitro adherence to intestinal cells and antimicrobial properties. *Res. Microbiol.* 152 167–173. 10.1016/S0923-2508(01)01188-3 11316370

[B14] Frece J. Kos B. Svetec I. K. Zgaga Z. Mrsa V. Suskovic J. (2005). Importance of S-layer proteins in probiotic activity of *Lactobacillus acidophilus* M92. *J. Appl. Microbiol.* 98 285–292. 10.1111/j.1365-2672.2004.02473.x 15659182

[B15] Granato D. Perotti F. Masserey I. Rouvet M. Golliard M. Servin A. (1999). Cell surface-associated lipoteichoic acid acts as an adhesion factor for attachment of *Lactobacillus johnsonii* La1 to human enterocyte-like Caco-2 cells. *Appl. Environ. Microbiol.* 65 1071–1077. 10049865 10.1128/aem.65.3.1071-1077.1999 PMC91146

[B16] Gueimonde M. Margolles A. de los Reyes-Gavilan C. G. Salminen S. (2007). Competitive exclusion of enteropathogens from human intestinal mucus by *Bifidobacterium* strains with acquired resistance to bile preliminary study. *Int. J. Food Microbiol.* 113 228–232. 10.1016/j.ijfoodmicro.2006.05.017 16842877

[B17] Jacobsen C. N. Nielsen V. R. Hayford A. E. Møller P. L. Michaelsen K. F. Paerregaard A. (1999). Screening of probiotic activities of forty-seven strains of *Lactobacillus* spp. by in vitro techniques and evaluation of the colonization ability of five selected strains in humans. *Appl. Environ. Microbiol.* 65 4949–4956. 10543808 10.1128/aem.65.11.4949-4956.1999 PMC91666

[B18] Johnson-Henry K. C. Hagen K. E. Gordonpour M. Tompkins T. A. Sherman P. M. (2007). Surface-layer protein extracts from *Lactobacillus helveticus* inhibit enterohaemorrhagic *Escherichia coli* O157:H7 adhesion to epithelial cells. *Cell. Microbiol.* 9 356–367. 10.1111/j.1462-5822.2006.00791.x 16925785

[B19] Kaila M. Isolauri E. Saxelin M. Arvilommi H. Vesikari T. (1995). Viable versus inactivated *Lactobacillus* strain GG in acute rotavirus diarrhoea. *Arch. Dis. Child.* 72 51–53. 10.1136/adc.72.1.51 7717739 PMC1510973

[B20] Kato I. Endo K. Yokokura T. (1994). Effects of oral administration of *Lactobacillus casei* on antitumor responses induced by tumor resection in mice. *Int. J. Immunopharmacol.* 16 29–36. 10.1016/0192-0561(94)90116-3 8150553

[B21] Lee Y. K. Puong K. Y. Ouwehand A. C. Salminen S. (2003). Displacement of bacterial pathogens from mucus and Caco-2 cell surface by lactobacilli. *J. Med. Microbiol.* 52 925–930. 10.1099/jmm.0.05009-0 12972590

[B22] Li P. C. Ye X. L. Yang Y. Q. (2011). Antagonistic activity of *Lactobacillus acidophilus* ATCC 4356 S-layer protein on *Salmonella enterica* subsp. enterica serovar Typhimurium in Caco-2 cells. *Ann. Microbiol.* 62 905–909. 10.1007/s13213-011-0327-1

[B23] Martinez B. Sillanpaa J. Smit E. Korhonen T. K. Pouwels P. H. (2000). expression of cbsA encoding the collagen-binding S-protein of *Lactobacillus crispatus* JCM5810 in *Lactobacillus casei* ATCC393. *J. Bacteriol.* 182 6857–6861. 10.1128/JB.182.23.6857-6861.2000 11073938 PMC111436

[B24] Neeser J. R. Granato D. Rouvet M. Servin A. Teneberg S. Karlsson K. A. (2000). *Lactobacillis Johnsonii* La1 shares carbohydrate-binding specificities with several enteropathogenic bacteria. *Glycobiology* 10 1193–1199. 10.1093/glycob/10.11.1193 11087711

[B25] Ouwehand A. C. Isolauri E. Kirjavainen P. V. ToÈlkkoÈ S. Salminen S. J. (2000). The mucus binding of *Bifidobacterium lactis* Bb12 is enhanced in the presence of *Lactobacillus* GG and *Lact. delbrueckii* ssp. *bulgaricus.* *Lett. Appl. Microbiol.* 30 10–13. 10.1046/j.1472-765x.2000.00590.x 10728552

[B26] Ouwehand A. C. Salminen S. (2003). In vitro adhesion assays for probiotics and their in vivo relevance: a review. *Microb. Ecol. Health Dis.* 15 175–184. 10.1080/08910600310019886

[B27] Ouwehand A. C. Salminen S. Tolkko S. Roberts P. Ovaska J. Salminen E. (2002). Resected human colonic tissue: new model for characterizing adhesion of lactic acid bacteria. *Clin. Diag. Lab. Immunol.* 9 184–186. 10.1128/cdli.9.1.184-186.2002 PMC119867 11777852

[B28] Ouwehand A. C. Salminen S. J. (1998). The health effects of cultured milk products with viable and non-viable bacteria. *Int. Dairy J.* 8 749–758. 10.1016/S0958-6946(98)00114-9

[B29] Papadimitriou K. Zoumpopoulou G. Foligné B. Alexandraki V. Kazou M. Pot B. (2015). Discovering probiotic microorganisms: in vitro, in vivo, genetic and omics approaches. *Front. Miocrobiol.* 6:58 10.3389/fmicb.2015.00058 PMC4330916 25741323

[B30] Perdigon G. Alvarez S. Gobbato N. de Budeguer M. V. de Ruiz Holgado A. A. P. (1995). Comparative effect of the adjuvant capacity of *Lactobacillus casei* and lipopolysaccharide on the intestinal secretory antibody response and resistance to *Salmonella* infection in mice. *Food Agric. Immunol.* 7 283–294. 10.1080/09540109509354886

[B31] Pham L. C. van Spanning R. J. Roling W. F. Prosperi A. C. Terefework Z. Ten Cate J. M. (2009). Effects of probiotic *Lactobacillus salivarius* W24 on the compositional stability of oral microbial communities. *Arch. Oral Biol.* 54 132–137. 10.1016/j.archoralbio.2008.09.007 18976742

[B32] Rojas M. Ascencio F. Conway P. L. (2002). Purification and characterization of a surface protein from *Lactobacillus fermentum* 104R that binds to porcine small intestinal mucus and gastric mucin. *Appl. Environ. Microbiol.* 68 2330–2336. 10.1128/AEM.68.5.2330-2336.2002 11976105 PMC127527

[B33] Roos S. Jonsson H. A. (2002). A high-molecular-mass cell-surface protein from *Lactobacillus reuteri* 1063 adheres to mucus components. *Microbiology* 148 433–442. 10.1099/00221287-148-2-433 11832507

[B34] Salminen S. Bouley C. Boutron-Ruault M. C. Cummings J. H. Franck A. Gibson G. R. (1998). Functional food science and gastrointestinal physiology and function. *Br. J. Nutr.* 80 S147–S171. 10.1079/bjn19980108 9849357

[B35] Salminen S. Ouwehand A. C. Benno Y. Lee Y. K. (1999). Probiotics: how should they be defined? *Trends Food Sci. Technol.* 10 107–110. 10.1016/S0924-2244(99)00027-8

[B36] Schiffrin E. J. Brassard D. Servin A. L. Rochat F. Donnet- Hughes A. (1997). Immune modulation of blood leukocytes in humans by lactic acid bacteria: criteria for strain selection. *Am. J. Clin. Nutr.* 66 515–520. 10.1093/ajcn/66.2.515S 9250141

[B37] Sillanpää J. Martínez B. Antikainen J. Toba T. Kalkkinen N. Tankka S. (2000). Characterization of the collagen-binding S-layer protein CbsA of *Lactobacillus crispatus*. *J. Bacteriol.* 182 6440–6450. 10.1128/JB.182.22.6440-6450.2000 11053389 PMC94791

[B38] Singh T. P. Kaur G. Malik R. K. Schillinger U. Guigas C. Kapila S. (2012). Characterization of Intestinal *Lactobacillus reuteri* strains as potential probiotics. *Probiotics Antimicrob. Proteins* 4 47–58. 10.1007/s12602-012-9090-2 26781736

[B39] Singh T. P. Malik R. K. Kaur G. (2016). Cell surface proteins play an important role in probiotic activities of *Lactobacillus reuteri*. *Nutrire* 41 5 10.1186/s41110-016-0007-9

[B40] Tareb R. Bernardeau M. Gueguen M. Vernou J. P. (2013). In vitro characterization of aggregation and adhesion properties of viable and heat-killed forms of two probiotic *Lactobacillus* strains and interaction with foodborne zoonotic bacteria, especially *Campylobacter jejuni*. *J. Med. Microbiol.* 62 637–649. 10.1099/jmm.0.049965-0 23329323

[B41] Van Tassell M. L. Miller M. J. (2011). *Lactobacillus* adhesion to mucus. *Nutrients* 3 613–636. 10.3390/nu3050613 22254114 PMC3257693

[B42] Wang B. Li Q. R. Li F. N. Luo N. Li Y. S. Li N. (2008). Isolation and identification of an adhesive probiotic *Lactobacillus* strian from human gastrointestinal tract. *Chin. J. Biol.* 21 0463–0466.

[B43] Weizman Z. Asli G. Alsheikh A. (2005). Effect of a probiotic infant formula on infections in child care centers: comparison of two probiotic agents. *Pediatrics* 115 5–9. 10.1542/peds.2004-1815 15629974

[B44] Wine E. Gareau M. G. Johnson-Henry K. Sherman P. M. (2009). Strain-specific probiotic (*Lactobacillus helveticus*) inhibition of *Campylobacter jejuni* invasion of human intestinal epithelial cells. *FEMS Microbiol. Lett.* 300 146–152. 10.1111/j.1574-6968.2009.01781.x 19765084

[B45] Zhang W. Wang H. Liu J. Zhao Y. Gao K. Zhang J. (2013). Adhesive ability means inhibition activities for *Lactobacillus* against pathogens and S-layer protein plays an important role in adhesion. *Anaerobe* 22 97–103. 10.1016/j.anaerobe.2013.06.005 23792230

